# Risk for life-threatening arrhythmia in newly diagnosed peripartum cardiomyopathy with low ejection fraction: a German multi-centre analysis

**DOI:** 10.1007/s00392-017-1090-5

**Published:** 2017-03-08

**Authors:** David Duncker, Ralf Westenfeld, Torsten Konrad, Tobias Pfeffer, Carlos A. Correia de Freitas, Roman Pfister, Dierk Thomas, Alexander Fürnkranz, René P. Andrié, Andreas Napp, Jörn Schmitt, Laszlo Karolyi, Reza Wakili, Denise Hilfiker-Kleiner, Johann Bauersachs, Christian Veltmann

**Affiliations:** 1 Department of Cardiology and Angiology, Hannover Medical School, Carl-Neuberg-Str. 1, 30625 Hannover, Germany; 2 Division of Cardiology, Pulmonology and Vascular Medicine, University Hospital Düsseldorf, Heinrich-Heine-University Düsseldorf, Düsseldorf, Germany; 3 II. Medical Clinic, Department of Electrophysiology, University Medical Center, Johannes Gutenberg-University, Mainz, Germany; 4 Lukaskrankenhaus Neuss, Neuss, Germany; 5 Department III of Internal Medicine, University of Cologne, Heart Center, Cologne, Germany; 6 Department of Cardiology, Medical University Hospital, Heidelberg, Germany; 7 Cardioangiologisches Centrum Bethanien, Medizinische Klinik III, Markus Krankenhaus, Frankfurt am Main, Germany; 8 Department of Internal Medicine II, Cardiology, University Hospital Bonn, Bonn, Germany; 9 Department of Internal Medicine I (Cardiology, Angiology, Pneumology and Internal Intensive Care Medicine), University Hospital, RWTH Aachen University, Aachen, Germany; 10 Department of Internal Medicine I, Division of Cardiology, University of Giessen, Giessen, Germany; 11 Praxisklinik Herz und Gefäße, Kardiologie-Angiologie-Radiologie-Nuklearmedizin, Dresden, Germany; 12 Department of Medicine I, University Hospital Munich, Campus Grosshadern, Ludwig-Maximilians-University, Munich, Germany

**Keywords:** Peripartum cardiomyopathy, Sudden cardiac death, Ventricular tachyarrhythmia, Wearable cardioverter/defibrillator

## Abstract

**Introduction:**

Peripartum cardiomyopathy (PPCM) is a rare cardiomyopathy characterized by an acute reduction in left ventricular ejection fraction (LVEF). Sudden deaths during the course of PPCM are reported to be elevated, the underlying mechanisms remains unknown. The aim of the present multi-centre study was to evaluate the arrhythmia burden in a multi-centre approach in patients with PPCM using a wearable cardioverter/defibrillator (WCD).

**Methods and results:**

Forty-nine patients from 16 German centres with newly diagnosed PPCM and LVEF ≤35% receiving a WCD were included in this retrospective analysis. Mean follow-up was 15 ± 10 months. At diagnosis, mean age was 33 ± 5 years, parity was 2.1 ± 1.6, LVEF was 21 ± 7%, NYHA functional class was 3.4 ± 0.7. Mean wear time was 120 ± 106 days, mean wear time per day was 21.4 ± 3.3 h. Six (12%) patients presented eight ventricular tachyarrhythmias during WCD period: five episodes of VF, two sustained ventricular tachycardia (VT) and one non-sustained VT occurred.

**Conclusion:**

This multicentre study underpins the elevated risk for ventricular tachyarrhythmias in patients with newly diagnosed PPCM and reduced LVEF. A WCD should be considered for 3–6 months in these patients to prevent sudden cardiac death from ventricular tachyarrhythmias.

## Introduction

Peripartum cardiomyopathy (PPCM) is a rare cardiomyopathy with acute, possibly severe and sometimes life-threatening course [1, 2]. Even though the rate of sudden deaths in the course of PPCM seems to be elevated [3], the underlying mechanisms are yet to be elucidated. Data on arrhythmia in PPCM is patchy [4]. One known mechanism of sudden death in left ventricular dysfunction is ventricular arrhythmia. In a small single-centre study, we recently reported that a relevant proportion of patients with newly diagnosed PPCM experiences life-threatening ventricular arrhythmias in the first months after diagnosis, thereby making arrhythmias the most plausible mechanism for sudden cardiac death (SCD) in this population [5]. Sudden arrhythmic death, however, may potentially be avoided by defibrillator therapy. Since the majority of patients with PPCM recover in LV dysfunction within 3–6 months, implantation of a permanent cardioverter/defibrillator for a transient time of risk would be an overtreatment.

The wearable cardioverter/defibrillator (WCD) represents a safe, non-invasive and effective option to prevent sudden arrhythmic death in patients with a transient or unknown risk [6]. Additionally, the WCD enables continuous rhythm monitoring especially for asymptomatic tachyarrhythmias. Therefore, the aim of the present multicentre study was to evaluate the arrhythmia burden in a multicentre approach in patients with PPCM and reduced LVEF using the WCD.

## Methods

In this national multi-centre study, patient data from 16 German primary, secondary and tertiary centres were pooled for retrospective analysis. The study was conducted in accordance with the Declaration of Helsinki. All patients with newly diagnosed PPCM and reduced left ventricular ejection fraction (LVEF) ≤35% receiving a WCD were included from each participating centre. Date of diagnosis was between 10/2011 and 03/2016. Data of 7 patients from Hannover Medical School have already been presented previously [5]. Diagnosis of PPCM was established according to the ESC definition [1]. All patients showed an LVEF ≤35% at diagnosis. Patients received standard heart failure therapy according to current guidelines and were followed-up according to the treating physician’s discretion. All patients received a WCD (LifeVest^®^, ZOLL, Pittsburgh, PA, USA) after diagnosis. WCD data (arrhythmia events, wearing compliance, technical problems) were registered with the remote monitoring system provided by the manufacturer (LifeVest Network^®^, ZOLL, Pittsburgh, PA, USA). Arrhythmias were classified by two experienced electrophysiologists (DD, CV) to assess the type of arrhythmia, the mode of onset and the mechanism of termination.

### Statistics

Continuous variables are presented as mean ± standard deviation and 95% confidence interval (CI). Categorical variables are presented as number of patients and percentages. Statistical analysis was performed using IBM SPSS Statistics version 24 (IBM, Armonk, NY, USA). Differences between groups were analysed using Student’s *t* test for continuous variables and Mann–Whitney *U* test for categorical variables, respectively. A *p* value <0.05 was considered statistically significant.

## Results

Forty-nine patients from 16 German centres were identified and fulfilled inclusion criteria. Date of diagnosis was between 10/2011 and 03/2016. Baseline characteristics are shown in Table 1A. No patient died during follow-up.

**Table 1:** Baseline characteristics (A) and wearable cardioverter/defibrillator (WCD) data (B) for all patients (*n* = 49)

	*n* = 49
*A. Baseline characteristics*	
Age (years)	33 ± 5 (95% CI 32–35)
Timing of diagnosis (days from delivery)	57 ± 57 (95% CI 41–73)
Parity (*n*)	2.1 ± 1.6 (95% CI 1.7–2.6)
LVEF at diagnosis (%)	21 ± 7 (95% CI 19–23)
NYHA functional class	3.4 ± 0.7 (95% CI 3.2–3.6)
NTproBNP (ng/L) (*n* = 37)	4965 ± 7328 (95% CI 2604–7327)
Betablocker	
*n*, %	98%)
% from target dose	43 ± 26 (95% CI 36–50)
ACE inhibitor/ARB	
*n*, %	98%)
% from target dose	40 ± 24 (95% CI 33–46)
MRA	
*n*, %	88%)
% from target dose	56 ± 44 (95% CI 44–68)
Bromocriptine	
*n*, %	43 (88%)
*B. WCD data*	
Wear time (days)	120 ± 106 (95% CI 90–150)
Wear time per day (h/day)	21.4 ± 3.3 (95% CI 20.5–22.2)
Patients with VT/VF episodes (*n*, %)	6 (12%)
Inappropriate WCD shocks (*n*)	0

*LVEF* left ventricular ejection fraction, *NYHA* New York Heart Association functional class, *ARB* angiotensin receptor blocker, *MRA* mineralocorticoid receptor antagonist, *VT* ventricular tachycardia, *VF* ventricular fibrillation

### WCD data

All patients received a WCD and were instructed to wear it continuously until re-evaluation. Cumulative wear time of WCD for all patients adds up to more than 15 patient-years (5838 days). WCD data are shown in Table 1B. One patient refused to continue wearing the WCD and further medical attendance after 10 days. However, she is known to be alive 12 months after diagnosis. Furthermore, another five patients showed a reduced wearing compliance of <20 h/day. One of them was unable to wear the WCD continuously because of a progressive dermatomycosis adjacent to the lateral shock electrode.

### Brady- and tachyarrhythmias

**Fig. 1:**
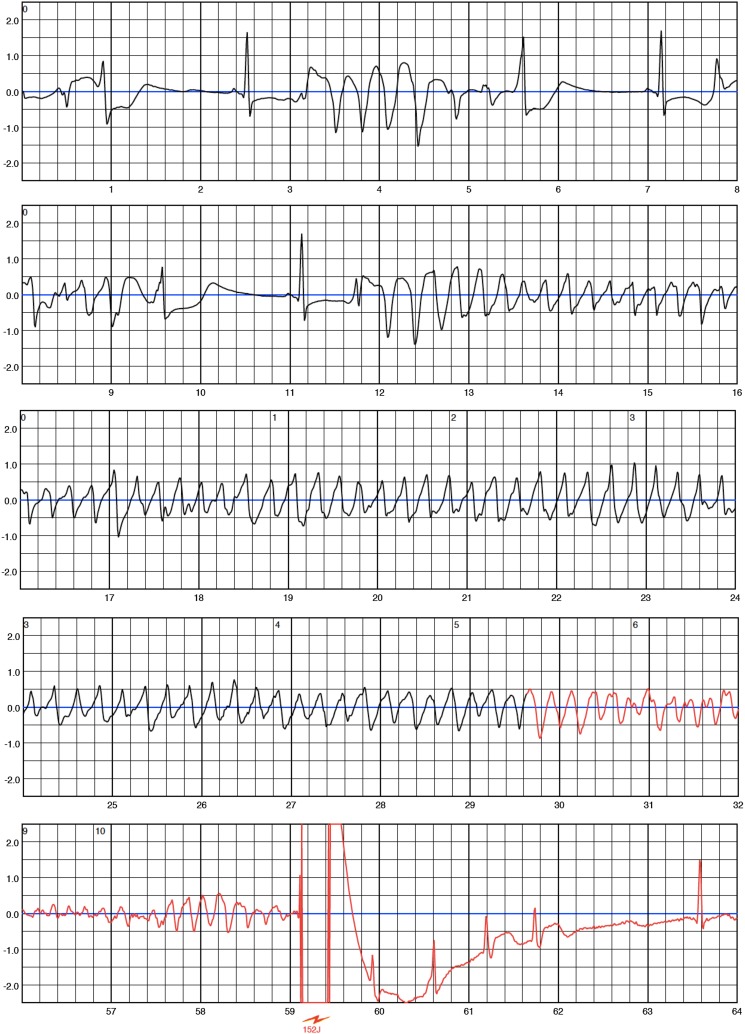
One episode of ventricular fibrillation (VF) in patient #11 with appropriate therapy by the wearable cardioverter/defibrillator. Note the short episodes of non-sustained ventricular tachycardia before onset of VF

**Fig. 2:**
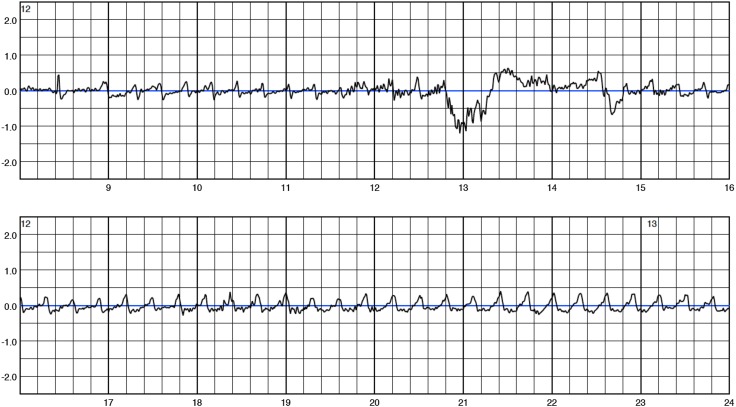
This figure depicts the onset of one episode of sustained monomorphic ventricular tachycardia (VT) in patient #42. Tachycardia cycle length was 290 ms (205bpm). The VT was hemodynamically tolerated for more than 20 min until cardioversion in the emergency room

**Fig. 3:**
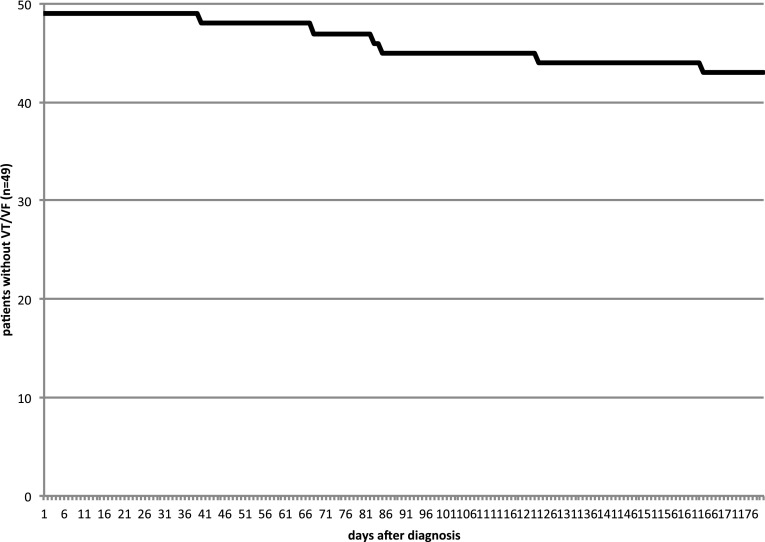
Incidence of ventricular tachyarrhythmias during wearable cardioverter/defibrillator period

No supraventricular arrhythmias or asystoles were detected. Eight ventricular arrhythmias were detected by the WCD in six patients during WCD wearing period. Five episodes of ventricular fibrillation (VF), two sustained ventricular tachycardia (VT) and one non-sustained ventricular tachycardia (nsVT) occurred (see Figs. 1, 2). Ventricular arrhythmias occurred between 30 and 160 days after diagnosis of PPCM (Fig. 3). Detailed information on arrhythmia episodes are shown in Table 2. One patient with 2 VF episodes within 1 h refused ICD implantation for a prolonged time and continued WCD wearing for more than 1 year, finally accepting ICD implantation. Another patient showed stable monomorphic VT (TCL 290 ms) after 160 days (Fig. 2), starting with a first episode terminating spontaneously after 2 min but starting again after a few minutes. The patient was able to call the ambulance and VT was hemodynamically tolerated during transport. The duration of sustained VT documented is more than 20 min. The patient withheld any WCD therapy by pushing the response buttons and WCD was taken off in the emergency room where cardioversion was performed. In this cohort, no inappropriate therapies were delivered by the WCD.

**Table 2:** Episodes of ventricular tachyarrhythmia documented by the wearable cardioverter/defibrillator (WCD). *TCL* tachycardia cycle length

Episode #	Patient #	Event	Time from diagnosis to event	Mechanism of induction	Termination	Time from detection to shock
1	7	Ventricular fibrillation	40 days	Spontaneous	Appropriate WCD shock	34 s
2	11	Ventricular fibrillation	68 days	Short-long-short sequence	Appropriate WCD shock	34 s
3		Ventricular fibrillation	68 days	Short-long-short sequence	Appropriate WCD shock	29 s
4	15	Ventricular fibrillation	83 days	Short-long-short sequence	Appropriate WCD shock	116 s
5	18	Ventricular fibrillation	124 days	Short-long-short sequence	Appropriate WCD shock	50 s
6	23	1 non-sustained ventricular tachycardia (TCL 380 ms)	85 days	Spontaneous	Spontaneous after 17 s	17 s (no shock)
7	42	Sustained ventricular tachycardia (TCL 290 ms)	165 days	Spontaneous	Spontaneous after 2 min	>2 min (no shock)
8		Sustained ventricular tachycardia (TCL 290 ms)	165 days	Spontaneous	WCD therapy withheld by patient, cardioversion in emergency room, VT duration >20 min	>20 min (cardioversion)

Comparison of baseline parameters did not show any significant differences between patients with vs. without ventricular arrhythmias (Table 3).

**Table 3:** Comparison of baseline parameters between patients with (*n* = 43) and without (*n* = 6) ventricular arrhythmias did not show significant differences

	Patients without ventricular arrhythmias (*n* = 43)	Patients with ventricular arrhythmias (*n* = 6)
Age (years)	34 ± 5 (95% CI 32–35)	31 ± 2 (95% CI 29–33)
Timing of diagnosis (days from delivery)	56 ± 57 (95% CI 39–73)	69 ± 54 (95% CI 26–113)
Parity (*n*)	2.1 ± 1.7 (95% CI 1.6–2.6)	2.3 ± 0.9 (95% CI 1.6–3.1)
LVEF at diagnosis (%)	21 ± 7 (95% CI 19–23)	19 ± 8 (95% CI 13–25)
NYHA functional class at diagnosis	3.4 ± 0.8 (95% CI 3.2–3.6)	3.2 ± 0.7 (95% CI 2.6–3.7)

### Follow-up

One patient was lost to follow-up after 10 days. For the remaining 48 patients, mean follow-up was 15 ± 10 (12–17) months. Thirty-nine (80%) patients showed recovery of LVEF beyond 45%. Mean LVEF at last follow-up was 48.5 ± 10.9 (45–52) %. NYHA functional class at last follow-up was 1.4 ± 0.6 (1.2–1.6). All patients with ventricular arrhythmic events finally received an ICD or CRT-D. The patient with the nsVT had a primary preventive ICD indication due to persistently reduced LVEF of 25%. Overall, at last follow-up, an ICD was implanted in seven patients and a CRT-D was implanted in four patients, respectively. After termination of the WCD wearing period, no further VT/VF episodes or syncopes were reported during long-term follow-up. One patient received ablation of monomorphic premature ventricular contractions. No ventricular tachyarrhythmia was documented in ICD/CRT-D recipients.

## Discussion

The present study was conducted to analyse the burden of ventricular arrhythmias in a larger population with newly diagnosed PPCM using the WCD. This is the largest study on patients with PPCM and severely reduced LVEF with continuous rhythm monitoring during the early phase of the disease.

The main finding of the study is that in a national, multi-centre approach, we found a relevant amount of VF episodes and potentially life-threatening ventricular arrhythmias in patients with newly diagnosed PPCM and reduced LVEF. During the WCD wearing period of about 3–6 months, we observed episodes of VF, sustained VT and nsVT in 12% of patients confirming an increased risk of arrhythmias in early PPCM.

Knowledge of aetiology, risk-factors and management of PPCM has strongly evolved in recent years [7]. However, the literature on the incidence of ventricular arrhythmias and sudden death in the course of PPCM is sparse. This refers to the rareness and also a presumed underdiagnosis of the disease. Thus, only case reports, case series and retrospective registries are available for estimation of the incidence of ventricular tachyarrhythmias and sudden death in PPCM [4]. Diao et al. described 1 out of 19 patients with PPCM presenting 4 nsVT during a 24 h Holter ECG [8]. In a case series from Pakistan, Laghari reported 3 (6.6%) patients with a VT at presentation [9]. A retrospective analysis of patients with PPCM wearing a WCD did not show any ventricular arrhythmias during WCD wearing [10]. However, specificity of the diagnosis of PPCM, especially in this study is disputable, since the diagnosis was only based on referral for cardiomyopathy coupled with a reference on the WCD-prescription to a current or recent pregnancy, thereby not representing the diagnostic criteria of the ESC. In a single-centre study, we have previously reported that there is a relevant risk for VF in the first months after diagnosis of PPCM with severely reduced left ventricular function [5]. However, a potential bias cannot be excluded as our centre is a tertiary centre with special focus on PPCM.

Thus, there was a need to put our previous results into perspective including patients with PPCM from Germany wearing a WCD after diagnosis. The present study is the first multicentre study representing patients with newly diagnosed PPCM treated in primary, secondary and tertiary medical centres. In all patients, the diagnosis of PPCM was based on the recommendation of the ESC [1].

In this multicentre study, we could underpin the high incidence of malignant ventricular tachyarrhythmias in 12% of the patients early after diagnosis of PPCM with a severely reduced LVEF. Thus, our multicentre data suggest a potential benefit from a temporary protection from SCD in this cardiomyopathy.

Patients with PPCM and severely reduced LVEF typically recover in the majority of cases [11]. Nevertheless, severely reduced LVEF remains at high risk for SCD. Our data shows that patients with PPCM are facing a relevant risk for life-threatening arrhythmias in the first months after diagnosis. The WCD has shown to be effective in preventing sudden cardiac death in several other cardiomyopathies [12], especially post myocardial infarction and in non-ischemic cardiomyopathy [13–15]. In the special setting of ICD removal due to infection, a WCD-guided strategy has been shown to be cost-effective [16]. An equivalent analysis for patients with PPCM having worn a WCD may support the decision to provide the WCD to these patients. Our data do not allow a cost-effectiveness analysis. However, saving young mother’s lives may be difficult to count up and costs of WCD- prescription may be justifiable.

In the early phase after diagnosis of a reduced LVEF, the WCD can offer protected time for optimization of heart failure medication and reverse left ventricular remodelling. A structured approach for prolonged but, however, secured optimization of heart failure therapies has been recently published [17].

The value of the WCD in our particular patients diagnosed with PPCM and reduced systolic LV function is the prevention of sudden arrhythmic death but without any long-term risks of an implantable device, especially considering the high rate of recovery. Since the events in our study occurred after 30–160 days after diagnosis, we suggest a wearing time of the WCD for 3–6 months independently of the LVEF as VF was observed in one patient even after recovery of LVEF to 45%.

## Limitations

Peripartum cardiomyopathy remains a rare disease and structured studies on arrhythmia burden have not been performed to date. The present study is a retrospective study and is therefore, prone for all known limitations of this study design. It may reflect a selection bias since only patients having worn a WCD were included. Only patients with LVEF ≤35% were prescribed a WCD in the participating centres, therefore, our highly selected cohort shows a much lower mean LVEF than other cohorts reported from Germany [11] or elsewhere [3]. As wear time in our cohort was excellent, WCD wearing serves as a long-term ECG monitor offering the chance to document symptomatic and asymptomatic ventricular tachyarrhythmias. Larger studies on world-wide incidence, management and outcome in PPCM are actually under way [18, 19]. These studies should aim to identify the mode of death and the arrhythmia burden, to improve prevention from sudden arrhythmic death in these patients.

## Conclusion

Patients with newly diagnosed PPCM and reduced LVEF show an elevated risk for ventricular tachyarrhythmias. A WCD should be considered for 3–6 months in all of these patients to prevent sudden cardiac death due to ventricular tachyarrhythmias.

## Appendix

### Participating sites

Department of Cardiology and Angiology, Hannover Medical School, Hannover, Germany: D. Duncker; Division of Cardiology, Pulmonology and Vascular Medicine, University Hospital Düsseldorf, Heinrich-Heine-University Düsseldorf, Düsseldorf, Germany: R. Westenfeld; II. Medical Clinic, Department of Electrophysiology, University Medical Center, Johannes Gutenberg-University, Mainz, Germany: T. Konrad; Lukaskrankenhaus Neuss, Neuss, Germany: C. A. Correia de Freitas; Department III of Internal Medicine, University of Cologne, Heart Center, Germany: R. Pfister; Department of Cardiology, Medical University Hospital, Heidelberg, Germany: D. Thomas; Cardioangiologisches Centrum Bethanien, Medizinische Klinik III, Markus Krankenhaus, Frankfurt am Main, Germany: A. Fürnkranz; Department of Internal Medicine II, Cardiology, University Hospital Bonn, Bonn, Germany: R. P. Andrié; Department of Cardiothoracic Surgery, Klinikum Augsburg, Augsburg, Germany: F. Dziewior; Department of Internal Medicine I (Cardiology, Angiology, Pneumology and Internal Intensive Care Medicine), University Hospital, RWTH Aachen University, Aachen, Germany: A. Napp; Department of Internal Medicine I, Division of Cardiology, University of Giessen, Gießen, Germany: J. Schmitt; Praxisklinik Herz und Gefäße, Kardiologie-Angiologie-Radiologie-Nuklearmedizin, Dresden, Germany; Department of Medicine I, University Hospital Munich, Campus Grosshadern, Ludwig-Maximilians-University Munich, Germany: C. Schuhmann, R. Wakili; Department of Cardiology, Heart and Diabetes Center North Rhine-Westfalia, Ruhr University Bochum, Bad Oeynhausen, Germany: K.-J. Gutleben; Department of Cardiology and Angiology, Augusta-Kranken-Anstalt, Bochum, Germany: E. Schulte; Department of Cardiology, Helios clinic Warburg, Warburg, Germany: S. Lindemann.
